# Prognostic Analysis of Pneumocystis Jirovecii Pneumonia in Interstitial Lung Disease Patients: A Retrospective Clinical Study

**DOI:** 10.3390/diagnostics12122925

**Published:** 2022-11-23

**Authors:** Yuxin Sun, Chi Shao, Hui Huang, Ruxuan Chen, Kai Xu, Mei Li, Xin Zhang, Zuojun Xu

**Affiliations:** 1 Department of Pulmonary and Critical Care Medicine, Peking Union Medical College Hospital, Chinese Academy of Medical Sciences & Peking Union Medical College, No. 1 Shuaifuyuan Street, Dongcheng District, Beijing 100730, China; 2 Department of Anesthesiology, China-Japan Friendship Hospital, No. 2 Yinghua East Street, Chaoyang District, Beijing 100029, China; 3 Radiological Department, Peking Union Medical College Hospital, Chinese Academy of Medical Sciences & Peking Union Medical College, No. 1 Shuaifuyuan Street, Dongcheng District, Beijing 100730, China; 4 Medical Records Department, Peking Union Medical College Hospital, Chinese Academy of Medical Sciences & Peking Union Medical College, No. 1 Shuaifuyuan Street, Dongcheng District, Beijing 100730, China

**Keywords:** Pneumocystis jirovecii pneumonia, human immunodeficiency virus negative, interstitial lung disease, prognosis

## Abstract

(1) Background: The clinical characteristics and the prognostic factors of HIV-negative Pneumocystis jirovecii pneumonia (PJP) patients (non-HIV-PJP) with interstitial lung disease (ILD) remain unclear. Our objectives were to describe the clinical characteristics and to explore the prognostic factors of non-HIV-ILD-PJP patients. (2) Methods: The enrolled patients in this retrospective study were stratified based on the presence or absence of ILD and fibrotic ILD (FILD). The log-rank test and Cox regression models were used to analyze the prognostic factors. (3) Results: Among 378 non-HIV-PJP patients, there were 133 patients with ILD-PJP, and 70 patients were classified as having FILD-PJP. The all-cause mortality rate for the ILD-PJP group is higher than that of the ILD-PJP group (57.9% vs. 38.4%, *p* < 0.001). However, the all-cause mortality is similar between the FILD-PJP group and non-FILD-PJP group. Preexisting ILD (HR: 2.156, *p* = 0.003) and honeycomb appearance on the chest HRCT (HR = 16.3, *p* < 0.001) are independent survival risk factors for ILD-PJP. Non-invasive ventilation is an independent survival risk factor for ILD-PJP patients (HR = 928.56, *p* < 0.01) and FILD-PJP patients (HR = 33.86, *p* < 0.001). (4) Conclusions: Pre-existing ILD and honeycomb appearance on the chest HRCT are independent survival risk factors for PJP patients. Non-invasive ventilation is associated with poor survival for both ILD-PJP and FILD-PJP patients.

## 1. Introduction

The prognosis of Pneumocystis jirovecii pneumonia (PJP) in human immunodeficiency virus (HIV) (HIV-PJP) has improved with the standardized management and prevention of HIV-PJP. However, non-HIV-PJP is still a serious disease [1]. The mortality rate of non-HIV-PJP remains high, with a reported rate of 30–60% [2]. A higher serum lactate dehydrogenase (LDH), older age, coinfection with cytomegalovirus (CMV), lower peripheral lymphocytes, pneumomediastinum, invasive ventilation, etc., are reported as associated risk factors for mortality in non-HIV-PJP patients [3,4,5,6]. Pre-existing chronic lung disease, especially interstitial lung disease (ILD), is a poor prognostic factor for PJP patients [7,8,9]. Corticosteroids and/or immunosuppressants are common medications for most ILD patients. These medications predispose non-HIV-infected patients to PJP [8,10]. On the other hand, the prevalence of Pneumocystis jirovecii (P. jirovecii) carriers is noted to be high in untreated ILD patients [11].

Diffuse ground-glass opacities (GGOs), consolidations, architectural distortions, fine reticulations, and/or traction bronchiolectasis are the classical chest high-resolution CT (HRCT) manifestations of PJP, and these manifestations might mimic the chest HRCT shadows that are seen in ILD patients [12,13,14,15,16,17]. Both PJP and ILD patients have similar symptoms, i.e., dry cough, dyspnea, and hypoxia. Therefore, it may be difficult to differentiate PJP from refractory ILD. PJP might be underdiagnosed or misdiagnosed. However, the delay in the initiation of anti-P. jirovecii treatment has been associated with the poor prognosis of non-HIV-PJP patients [18]. Few clinical studies to date have focused on exploring the prognostic factors for non-HIV-PJP in ILD patients (non-HIV-ILD-PJP).

Therefore, we conducted this retrospective analysis of hospitalized non-HIV-ILD-PJP patients to elucidate their clinical characteristics and their prognostic associated factors. We hypothesized that pre-existing ILD would be a risk factor for the survival of non-HIV-PJP patients.

## 2. Materials and Methods

All patients who were discharged with a diagnosis of PJP from January 2014 to December 2020 were screened for eligibility from the electronic medical records system at Peking Union Medical College Hospital (PUMCH).

Their complete medical records and radiological imaging data were retrospectively reviewed through the hospital’s electronic banks. They were followed up from the day that the diagnosis of PJP was confirmed. After discharge, they were followed up every 1 to 6 months, depending on the disease activity and severity. The final follow-up point was 30 June 2021. Follow-up information was obtained through outpatient records or telephone interviews. The survival time was defined as the time (in days) between the diagnosis of PJP to the date of death or the last follow-up point. The following information was reviewed and analyzed: age, sex, clinical manifestations, serological results, chest HRCT images, treatments, and outcomes.

The inclusion criteria included the following: (1) confirmed PJP diagnosis and (2) a negative HIV test. The exclusion criteria included the following: (1) an active PJP infection in the respiratory samples was not confirmed in the respiratory samples; (2) the patient was less than 18 years old or was pregnant; (3) the patient had a positive HIV test; and (4) the patient’s complete clinical, chest HRCT, and/or follow-up data were missing.

The criteria for the diagnosis of confirmed PJP were as follows: (1) the presence of relevant respiratory manifestations, i.e., cough, dyspnea, and/or hypoxia; (2) the presence of pulmonary shadows that were identified by chest HRCT; (3) identification of active P. jiroveci infection i.e., i. positive P. jiroveci DNA fragments in the respiratory samples, which was confirmed by polymerase chain reaction (PCR) and a serum 1,3-β-D-glucan (βDG) > 100 pg/mL, and/or ii. P. jirovecii asci were observed at direct microscopic examination with Grocott’s methenamine silver (GMS) stain in the respiratory samples. The respiratory samples included bronchoalveolar lavage fluid (BALF), aspirates, sputum, or lung tissue.

**Figure 1:**
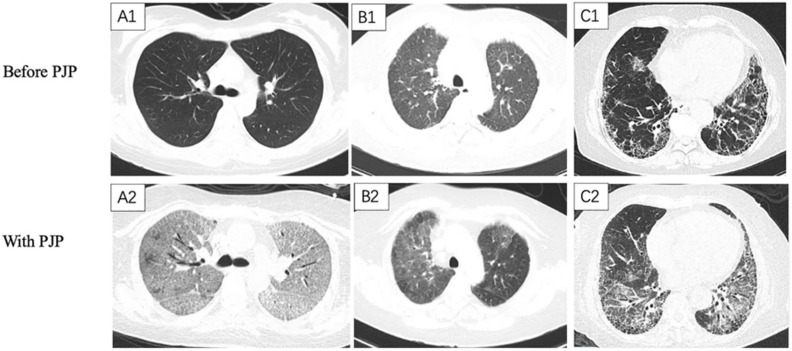
Chest CT scanning of Pneumocystis jirovecii pneumonia (PJP) with different underlying pulmonary diseases: (1) (**A1**,**A2**) A 52 year old female nephrotic syndrome patient without underlying pulmonary disease suffered from PJP 3 months later. (**A1**) Chest CT scan (16 April 2019) shows normal lung parenchymal before the administration of prednisone and mycophenolate mofetil. (**A2**) Chest CT scan (11 July 2019) obtained 3 months later showing diffuse ground-glass opacities (GGOs) and multiple traction bronchiectasis. She suffered from fever and dyspnea on July 2, 2019. She was diagnosed with PJP on 12 July 2019. (2) (**B1**,**B2**) A 36 year old female eosinophilic granulomatosis with polyangiitis patients with non-fibrosing interstitial lung disease was complicated with PJP 2 months later. (**B1**) Chest CT scan (8 March 2017) show diffused GGOs throughout the bilateral lungs before the administration of prednisone and iv. cyclophosphamide. (**B2**) Chest CT scan (5 May 2017) obtained 2 months later shows relapsing diffused GGOs in the right upper lobe and local GGOs in the left upper lobe. She complained of fever and dyspnea on 1 May 2017. She was diagnosed with PJP on 9 May 2017. (3) (**C1**,**C2**) A 60 year old male rheumatoid arthritis patients with fibrotic interstitial lung disease (FILD) was complicated with PJP 3 months later. (**C1**) Chest CT scan (11 May 2019) shows bilateral subpleural with basal predominance of the reticulation, interlobular septal thickening, and scattered honeycombs before the administration of Tofacitinib. (**C2**) Chest CT scan (8 August 2019) obtained 3 months later shows diffused GGOs and multiple traction bronchiectasis in the background of FILD. He had suffered from exaggerated dyspnea since August 3, 2019. He was diagnosed with PJP on 11 August 2019.

ILD was defined by the presence of hallmark manifestations on chest HRCT [14]. Fibrotic ILD (FILD) was diagnosed with a pre-PJP chest HRCT, which showed traction bronchiolectasis, reticulation, architectural distortion, lung volume loss, and/or honeycombs [19], and the chest CT patterns were in accordance with the usual interstitial pneumonia (UIP) pattern, probable UIP pattern, possible UIP pattern, fibrosing nonspecific interstitial pneumonia (NSIP) pattern [20], or fibrotic hypersensitivity pneumonitis (HP) pattern [21]. The findings in the PJP and pre-PJP HRCTs were reviewed by two pulmonologists (C.S. and H.H.) and one radiologist (K.X.). They were blinded to each other’s interpretations. The typical HRCT findings for non-ILD-PJPs, non-fibrotic ILD-PJPs, and FILD-PJPs are listed in Figure 1.

This study was approved by the Ethics Committee of Peking Union Medical College Hospital (JS-2517) in accordance with the Declaration of Helsinki. Our study was performed using anonymized health care data. The written informed consent from each patient was waived as this study met the Peking Union Medical College Hospital IRB’s minimal risk waiver criteria.

## 3. Statistical Analysis

The data were analyzed using the SAS version 9.4 software package (SAS Institute Inc., SAS Campus Drive, Cary, NC 27513, USA.). The quantitative variables are presented as the means ± standard deviations (SD), and the categorical data are presented as frequencies and percentages. The *t* test or rank sum test was used for measured data, and the chi-square test was used for count data. The difference was statistically significant when *p* < 0.05. The log-rank test was used to compare the survival rates of different subgroups, and Kaplan–Meier survival curves were plotted. Cox regression models were used to identify factors associated with mortality for PJP with different pre-existing statuses. The statistically significant variables selected via univariate analysis were finally assessed by multivariate analysis.

## 4. Results

### 4.1. General Characteristics of the Enrolled PJP Patients

**Figure 2:**
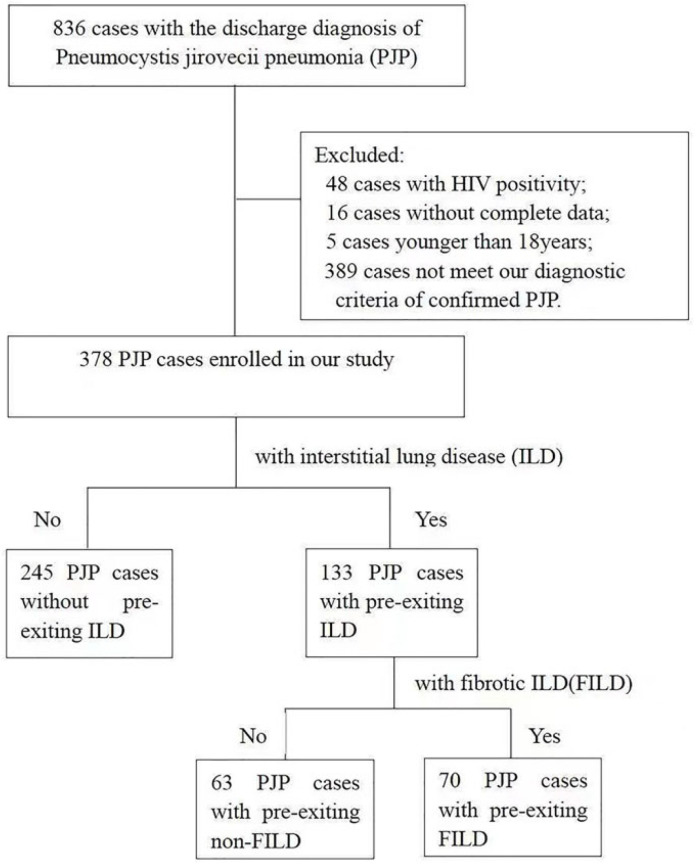
The study flow chart.

After a detailed medical records review, 836 PJP patients were enrolled in our study. Among them, there were 48 HIV patients, 5 patients younger than 18 years, 16 patients without complete clinical-radiological-follow-up data, and 389 patients without a confirmed PJP diagnosis according to our detailed diagnostic criteria. Three hundred and seventy-eight patients with PJP were included and were evaluated for the final analysis. Among them, there were 133 PJP cases with pre-existing ILD and 245 PJP cases without pre-existing ILD. The study flow chart is shown in Figure 2.

Among the 378 enrolled PJP cases, there were 172 males and 206 females, who were 54.0 ± 16.1 years old (range from 18 y to 88 y). Most of them had different underlying diseases (Table 1). The mean follow-up period was 371.1 days, which ranged from 2 to 2548 days. Hypoalbuminemia was common in the PJP patients (only two of the patients did not have serum albumin levels measured during PJP). There were 371 patients (98.7%) who had hypoalbuminemia, and the minimal serum albumin was 23.4 ± 5.1 g/L (range from 10 to 34 g/L, 325 cases/86.4% < 30 g/L). Blood glucose was tested in all 378 patients during PJP. There were 110 patients who were diagnosed with diabetes mellitus before PJP; however, more than half of the enrolled PJP patients (250/66.1%) showed hyperglycemia during PJP.

Most PJP patients were given corticosteroids and/or immunosuppressants before PJP: corticosteroids were given in 310 patients/82% (prednisone daily dosage > 1 mg/kg/d 266 patients/70.4%, methylprednisolone 500–1000 mg/d in 58 patients/15.3%); immunosuppressants were given in 233 patients/61.6% (including cyclophosphamide, tacrolimus, mycophenolate mofetil, etc.), and biological agents were given in 23/6.1% (including anti-tumor necrosis factor, rituximab, etc.). The management before PJP for 40 patients with malignancy included the following: chemotherapy (19/47.5%), chemotherapy and radiotherapy (4/10%), chemotherapy and immunotherapy (2/5%), chemotherapy, radiotherapy, and immunotherapy (1/1.25%), EGFR-TKI (1/1.25%), and radiotherapy with no medical therapy (14/35%).

Some of the PJP patients were co-infected with cytomegalovirus (CMV, 210/55.6%), common HAP pathogens (114/30.2%), different species of aspergilli (58/15.3%), oral candidiasis (37/9.8%), different species of mycobacteria (18/4.8%), and nocardia (7/0.2%).

Respiratory failure was defined as a room air pulse oxygenation of <90% or a room air arterial partial pressure oxygen level of <60 mmHg. There were 286 patients with PJP who suffered complications with respiratory failure during hospitalization, and 192 severe patients were transferred to the ICU wards because of high index hospitalization. Among them, 169 patients (44.7%) were supported with invasive ventilation, and 111 patients (29.4%) were supported with noninvasive ventilation. The overall mortality rate for all of the enrolled patients was 45.2%. Half of them (51.5%) died within 30 days after the diagnosis of PJP. A total of 84.2% of patients died within 60 days after PJP, and 93.0% of patients died within 3 months of the diagnosis of PJP.

A Cox regression multivariate analysis for PJP patients shows that pre-existing ILD (HR:2.156, χ^2^ = 2.96, *p* = 0.003, 95% CI: 1.30–3.59) and honeycombs in the chest HRCT (HR = 16.3, χ^2^ = 16.8, *p* < 0.001, 95% CI: 4.3–62) are the significant and independent risk factors for the mortal PJP patients. However, respiratory failure (HR = 0.001, χ^2^ = 7.6, *p* = 0.006, 95% CI: 0–0.13), hyperglycemia (HR = 0.004, χ^2^ = 11.2, *p* < 0.001, 95% CI: 0–0.1), decreased Ly% (HR = 0.003, χ^2^ = 9.1, *p* < 0.001, 95% CI: 0–0.05), serum albumin_min_ (HR = 0.32, χ^2^ = 13.3, *p* < 0.001, 95% CI: 0.18–0.59), consolidation in the chest HRCT (HR = 0.01, χ^2^ = 15.9, *p* < 0.001, 95% CI: 0–0.20), and pleural effusion (HR = 0.13, χ^2^ = 4.4, *p* = 0.04, 95% CI: 0.02–0.88) are protective factors for them. Therefore, the PJP patients with pre-existing ILD and FILD were analyzed in detail in the following study.

### 4.2. ILD-PJP vs. Non-ILD-PJP

The clinical manifestations, laboratory tests, radiological features, treatments, and prognostic factors of the ILD-PJP group (133 cases) versus the non-ILD-PJP group (245 cases) are described in Table 2. The underlying causes for ILDs were variable, including idiopathic inflammatory-myositis-associated ILD (36 cases), idiopathic interstitial pneumonia (24 cases), systemic vasculitis-associated ILD (19 cases), rheumatoid-arthritis-associated ILD (15 cases), systemic lupus-erythematosus-associated ILD (12 cases), interstitial pneumonia with autoimmune features (12 cases), radiation-induced lung injury (5 cases), primary Sjogren’s-syndrome-associated ILD (5 cases), drug-induced lung injury (4 cases), and systemic scleroderma-associated ILD (1 case). The non-ILD-PJP group was younger than ILD-PJP: 51.5 ± 16.7 years vs. 58.8 ± 13.9 years. The non-ILD-PJP group was more likely to have corticosteroid therapy (211/86.1% vs. 9/74.4%, *p* = 0.005); however, the ILD-PJP group was more likely to have a higher dose of corticosteroid medication (≥500 mg/d methylprednisolone) (29/21.8% vs. 29/11.8%, *p* = 0.01).

A low peripheral lymphocyte percentage (<20%) is common in both the ILD-PJP and non-ILD-PJP groups (89.5% vs. 85.2%). The lymphocyte percentage and the absolute lymphocyte count are [(9.4 ± 7.8)% vs. (11.7 ± 10.9)%] and [(0.79 ± 0.82) × 109/L vs. (0.86 ± 0.89) × 109/L] for the ILD-PJP vs. non-ILD-PJP groups, respectively. There were 94 patients/70.7% with ILD-PJP and 150 patients/61.5% with non-ILD-PJP who had lymphocytopenia. The serum immunoglobin (Ig)G level is lower in the non-ILD-PJP group [(8.4 ± 5.6) g/L vs. (10.3 ± 5.6) g/L, *p* < 0.001], and there are more patients in the non-ILD-PJP group with hypo-IgG (52.6% vs. 35.3%, *p* = 0.003).

The T-lymphocyte subset analysis is available for most of the enrolled PJP cases (Table 3): 111 patients (83.5%) with ILD-PJP and 205 patients (83.7%) with non-ILD-PJP. The percentages of B cells, NK cells, and CD4^+^ T cells are lower than normal among almost half of the enrolled PJP cases; however, the percentages of CD3^+^ T cells and CD8^+^ T cells are normal for most of the enrolled PJP patients. There is no significant difference between the ILD-PJP and non-ILD-PJP groups in the abnormal peripheral lymphocyte subset analysis.

ILD-PJP patients are more likely to suffer from pneumomediastinum than non-ILD-PJP patients (19.6% vs. 8.6%, *p* = 0.002). Although ILD-PJP patients are more likely to suffer with complications from respiratory failure than non-ILD-PJP patients (85.7% vs. 70.2%, *p* < 0.001), the requirements for ICU admission, invasive ventilation, and non-invasive ventilation are similar in the ILD-PJP group and the non-ILD-PJP group (48.9% vs. 51.8%, 45.1% vs. 44.5%, 29.3% vs. 29.4%, respectively, *p* > 0.05 for all). The interval between the onset and the diagnosis of ILD-PJP patients is longer than that of non-ILD-PJP patients [(31.1 ± 94.9) days vs. (21.0 ± 75.1) days, *p* = 0.047].

**Figure 3:**
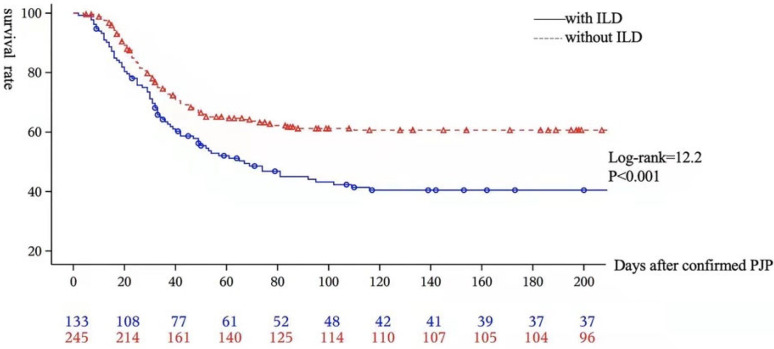
Kaplan–Meier survival analysis for all enrolled PJP patients. The all-cause mortality rate for the PJP in interstitial lung disease (ILD-PJP) group is higher than that of the non-ILD-PJP group (log-rank = 12.2, *p* < 0.001).

A Kaplan–Meier survival analysis and a Cox regression multivariate analysis were performed to identify the potential confounding prognostic factors with hospital mortality for ILD-PJP patients (Figure 3, Table 4). The all-cause mortality rate for the ILD-PJP group is higher than that of the non-ILD-PJP group. Approximately half of the PJP patients, both in the ILD-PJP group (49.4%) and the non-ILD-PJP group (53.2%), died within 30 days after the diagnosis of PJP, and more than 90% of the PJP patients died within 3 months after PJP (90.9% ILD-PJP patients vs. 96.7% non-ILD-PJP patients). Non-invasive ventilation (HR = 928.56, χ^2^ = 7.3, *p* < 0.01, 95% CI: 6.6–130,796.5) and serum D-dimer (HR = 1.2, χ^2^ = 10.9, *p* < 0.001, 95% CI: 1.01–1.34) are the significant independent risk factors for non-survival of ILD-PJP patients.

### 4.3. FILD-PJP vs. Non-FILD-PJP

**Figure 4:**
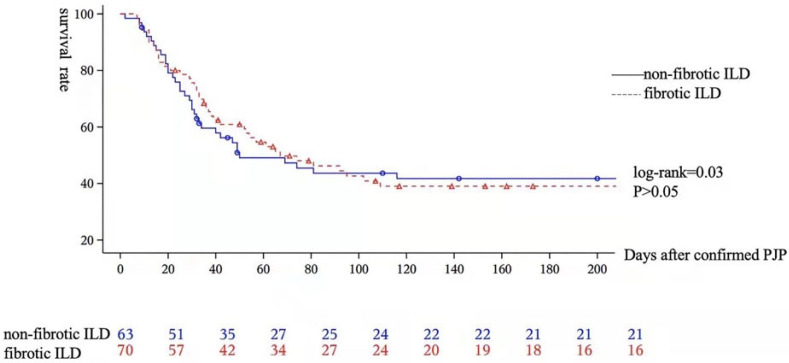
Kaplan–Meier survival analysis for all enrolled ILD-PJP patients. The all-cause mortality is similar between the FILD-PJP patients and the non-FILD-PJP patients (log-rank = 0.03, *p* > 0.5).

After reviewing the chest HRCT, the ILD-PJP patients were subdivided into the FILD-PJP group (70 patients) and the non-FILD-PJP group (63 patients). The clinical, laboratory, radiological, and prognostic characteristics were compared between them (Table 5). Invasive ventilation support is more likely to be performed for non-FILD-PJP patients; however, non-invasive ventilation is more likely to be performed for FILD-PJP patients. Both serum LDH and βDG are higher in non-FILD-PJP patients. The non-FILD-PJP patients are more likely to show elevated serum βDG than the FILD-PJP patients. However, the all-cause mortality is similar between them (Figure 4). The Cox regression multivariate model indicates that non-invasive ventilation is the significant independent risk factor for non-survival of FILD-PJP patients (HR:33.86, χ^2^ = 3.52, *p* < 0.001, 95% CI: 5.28–217.08).

## 5. Discussion

In our study, we find that pre-existing ILD, and honeycombs on the chest HRCT are the independent risk factors for non-survival of the PJP patients. Non-invasive ventilation is an independent survival risk factor for both ILD-PJP and FILD-PJP patients. The serum IgG level is lower in the non-ILD-PJP patients, and there are more non-ILD-PJP patients with hypo-IgG than ILD-PJP patients. ILD-PJP patients are more likely to suffer from complications with pneumomediastinum and respiratory failure than non-ILD-PJP patients. The all-cause mortality rate of ILD-PJP patients is higher than that of non-ILD-PJP patients. However, there is no significant difference in the all-cause mortality rate between the FILD-PJP and non-FILD-PJP patients.

Corticosteroids are the most commonly reported risk factors for non-HIV-PJP, but the association of the daily dose threshold, the cumulative dose, and the types of corticosteroids used for PJP is still unknown [10]. In our study, although there are more non-ILD-PJP patients administered corticosteroids, methylprednisolone pulse therapy is more common in ILD-PJP patients before PJP. Almost all of our PJP patients have hypoalbuminemia and hyperglycemia during PJP, regardless of the presence of basic diabetes mellitus. With the resolution of PJP, the serum albumin of the patients gradually increases to normal. Decreased serum albumin is reported as a poor prognostic factor for PJP [23,24,25], and as a risk factor for acute lung injury/acute respiratory distress syndrome [26]. The administration of treatment for diabetes mellitus is associated with PJP for kidney transplantation patients, but is not a prognostic factor [27]. However, both hyperglycemia and serum minimal albumin values are independent protective factors for PJP patients in our study. Hypoalbuminemia has been a major focus since Chen’s and Li’s studies in our hospital [25,28]. The active treatment of the abnormal blood glucose levels and hypoalbuminemia in our PJP patients might contribute to this association.

Although peripheral lymphocytopenia, especially a low CD4^+^ T-cell count, is a recognized risk factor for HIV-PJP, the peripheral lymphocyte percentage, absolute lymphocyte count, and subtype features are variable among the different studies of non-HIV-PJP patients administered corticosteroids [4,10,29,30,31]. A low peripheral lymphocyte percentage is more common than a low lymphocyte count for our PJP patients, which might be caused by the elevation of the WBC count after corticosteroid administration. There are few prospective studies that evaluate lymphocyte subtype analysis for non-HIV-PJP patients, and the lymphocyte subtype characteristics vary significantly among the different studies [32,33]. The percentages of CD3^+^ T cells and CD8^+^ T cells are normal for most of our enrolled PJP patients. This might be associated with the different pre-existing diseases and previous medications that the patients had before PJP.

There are several reported diagnostic and/or prognostic serum biomarkers for PJP patients. Serum LDH levels could be an adjunctive diagnostic biomarker for non-HIV-PJP, and elevated serum D-dimer and C-reactive protein and lymphopenia are reported to be associated with the prognosis of PJP patients [10,34,35]. Elevated serum D-dimer and/or fibrinogen are a concern in coronavirus disease 2019 (COVID-19) and other severe viral pneumonia diseases [36,37]. Severe pulmonary inflammation might trigger the activation of, and cause damage to, the pulmonary endothelia and can cause thrombosis during severe pneumonia. Many of our enrolled PJP patients presented with respiratory failure and/or severe hypoxia. Elevations in D-dimer and fibrinogen were common in both ILD-PJP and non-ILD-PJP patients. Both the serum LDH and βDG are higher in non-FILD-PJP patients than in FILD-PJP patients. Furthermore, elevations in D-dimer might be associated with the poor prognosis of ILD-PJP patients in our study. Hyperinflammatory characteristics, including elevations in ESR, CRP, D-dimer, and/or fibrinogen, should be paid attention to if they are present in ILD-PJP patients, as they might be poor prognostic factors.

The HRCT manifestations of PJPs were similar to those of ILDs [12,13,14,15], and it was more difficult to differentiate PJP from the worsening of a pre-existing ILD in ILD-PJP patients than in non-ILD-PJP patients. Extensive diffuse GGO is the main CT feature for early PJP, and it is usually symmetric and apical, and has a perihilar distribution [12,13]. Reversible architectural distortions, including irregular linear opacities, reticulations, and traction bronchiolectasis, appear during the follow-up chest CT scans in the non-HIV-PJP patients. A variable amount of pulmonary fibrosis could be beneficial for HIV-PJP patients; however, it rarely occurs in non-HIV-PJP patients [13]. GGOs, reticulation, traction bronchiolectasis, and honeycombs are also common chest manifestations of ILD [14,15,38]. Therefore, the complications associated with PJP might be neglected or misdiagnosed in ILD patients. Concomitant ILD is associated with a poor prognosis for non-immunocompromised PJP patients [39]. The interval between the onset and the diagnosis of the ILD-PJP patients is longer than that of the non-ILD-PJP patients in our study. It is also reported that a delayed diagnosis and the initiation of anti-PJP treatment are risk factors for mortality in non-HIV-PJP patients [18]. PJP patients should be monitored for ILDs, especially patients who are administered corticosteroids and/or immunosuppressants.

The all-cause mortality rate of ILD-PJP patients is higher than that of non-ILD-PJP patients in our study. Co-infection with aspergillosis, honeycombs in the chest HRCT, invasive ventilation, and non-invasive ventilation are significantly independent risk factors for mortal PJP patients. The application of mechanical ventilation, decreased serum albumin, co-infection with aspergillosis or CMV, and underlying chronic pulmonary disease are reported as poor prognostic factors for PJP patients [4,5,24,40]. Few studies focused on the fibrosing characteristics on chest CTs in PJP patients. We found that the presence of honeycombs is a poor prognostic factor for non-HIV-PJP patients. It is necessary to pay attention to ILD-PJP patients who have honeycombs. Fortunately, although honeycombs are an independent risk factor for non-survival in ILD-PJP patients, there are no significant differences in the all-cause mortality rate between the FILD-PJP patients and the non-FILD-PJP patients.

There are several limitations in our retrospective study. First, all patients were diagnosed with confirmed PJP and were admitted to a tertiary hospital, which would lead to selection bias. Second, it was a retrospective study and almost more than half of the enrolled PJP cases were severe patients who were admitted or transferred to the ICU. Therefore, much information could not be collected completely and homologously, especially during the interval period of repeated chest HRCT frequencies. Third, although all enrolled PJP cases were administrated adjunctive corticosteroids, the detailed adjunctive corticosteroid protocol, especially the tapering protocol was not the same because of the various underlying diseases. A multicenter, well-designed prospective study is expected in the future.

## 6. Conclusions

Pre-existing ILD and honeycomb in the chest HRCT are independent survival risk factors for PJP patients. Non-invasive ventilation is an independent survival risk factor for both ILD-PJP and FILD-PJP patients. The all-cause mortality rate of ILD-PJP patients is higher than that of non-ILD-PJP patients. However, there is no significant difference in the all-cause mortality rate between the FILD-PJP and non-FILD-PJP patients.

## Figures and Tables

**Table 1:** The underlying diseases for enrolled PJP cases.

Underlying disease (*n* = 374)
Connective tissue diseases (CTDs, *n* = 184)
Systemic vasculitis (*n* = 48)
Systemic lupus erythematosus (*n* = 45)
Idiopathic inflammatory myositis (*n* = 40)
Rheumatoid arthritis (*n* = 17)
Primary Sjögren syndrome (*n* = 12)
Other kinds of CTDs (*n* = 22)
Kidney diseases (*n* = 59)
Primary glomerulonephritis (*n* = 56)
Renal failure (*n* = 3)
Malignancies (*n* = 58)
Hematological malignancy (*n* = 33)
Other malignancy (*n* = 25)
Pulmonary diseases (*n* = 28)
Idiopathic interstitial pneumonia (*n* = 24)
Emphysema (*n* = 3)
Sarcoidosis (*n* = 1)
Non-malignant hematological diseases (*n* = 18)
Dermatologic diseases (*n* = 13)
Neurological diseases (*n* = 4)
Transplantations (*n* = 4)
Endocrinologic diseases (*n* = 3)
Inflammatory bowel diseases (*n* = 2)
Severe drug-induced liver injury (*n* = 1)

PJP: Pneumocystis jirovecii pneumonia, CTD: connective tissue disease.

**Table 2:** The characteristics of ILD-PJP patients vs. non-ILD-PJP patients.

Variables	ILD-PJP (*n* = 133)	Non-ILD-PJP (*n* = 245)	t or χ^2^	*p* Value
Age (mean ± SD)y	54.1 ± 16.3	63.2 ± 9.6	4.17	<0.001
Gender (male, *n*/%)	67/50.4%	105/42.9%	1.97	0.16
Glucocorticosteroids before PJP (*n*/%)	99/74.4%	211/86.1%	7.98	<0.01
Prednisone (>1 mg/kg/d)	99/76.7%	167/72.9%	0.63	0.43
Methylprednisolone (>500 mg/d)	29/21.8%	29/11.8%	6.59	0.01
Respiratory failure (*n*/%)	114/85.7%	172/70.2%	6.54	0.01
Ventilation (*n*/%)				
Invasive ventilation	60/45.1%	109/44.5%	0.01	0.91
Non-invasive ventilation	39/29.3%	72/29.4%	0.001	0.99
ICU admission (*n*/%)	65/48.9%	127/51.8%	0.3	0.58
Pneumomediastinum (*n*/%)	26/19.56%	21/8.6%	9.54	<0.01
Death during PJP (*n*/%)	77/57.9%	94/38.4%	13.3	<0.01
Died from respiratory failure	45/33.8%	48/19.6%	3.33	<0.01
CMV viremia	76/57.1%	134/54.7%	0.21	0.65
Laboratory tests				
WBC (10^9^/L)(mean ± SD)	14.3 ± 66.6	9.66 ± 20.1	0.79	0.44
Lymphocyte (%)	9.4 ± 7.8	11.7 ± 10.9	1.66	0.1
Lymphocyte count (10^9^/L)(mean ± SD)	0.8 ± 0.8	0.9 ± 0.9	1.23	0.22
ESR elevation (*n*/%)	97/78.9%	149/76%	0.35	0.56
hsCRP elevation (*n*/%)	112/88.2%	180/83.0%	1.71	0.19
LDH (U/L) (mean ± SD)	614.4 ± 530	580.8 ± 351.0	0.75	0.46
LDH elevation (*n*/%)	120/94.5%	206/90.4%	1.86	0.17
Hypo-immunoglobin G %(*n*/%)	41/35.3%	100/52.6%	8.66	0.003
D-dimer(mg/L)	9.1 ± 21.8	7.9 ± 19.2	0.21	0.83
Fbg(g/L)	4.0 ± 1.9	4.3 ± 2.5	1.54	0.12
βDG(pg/mL)	692.5 ± 963.3	1129 ± 1266.7	4.47	<0.001
Chest HRCT features				
Sub-pleural distribution (*n*/%)	68/51.3%	48/19.6%	40.3	<0.001
Peri-bronchial distribution (*n*/%)	13/9.8%	22/9.0%	0.06	0.8
GGO (*n*/%)	106/79.7%	176/71.8%	2.81	0.09
Consolidation (*n*/%)	36/27.1%	106/43.3%	9.64	0.002
Reticular (*n*/%)	50/37.6%	17/6.9%	55.5	<0.001
Honeycomb (*n*/%)	19/14.3%	1/0.4%	33.1	<0.001
Nodular (*n*/%)	46/34.6%	112/45.7%	4.39	0.036
Septal thickening (*n*/%)	39/29.3%	16/6.5%	36.0	<0.001
Pleural thickening (*n*/%)	92/69.2%	120/49.0%	14.3	<0.001
Pleural effusion (*n*/%)	43/32.3%	101/41.2%	2.89	0.09
Lymphadenopathy (*n*/%)	79/59.4%	97/39.6%	13.6	<0.001
The interval between onset and diagnosis of PJP (days)	31.1 ± 94.9	21.0 ± 75.1	1.98	0.047

PJP: pneumocystis jiroveci pneumonia, ILD: interstitial lung disease, CMV: cytomegalovirus, Hb: hemoglobin, LDH: lactate dehydrogenase, Fbg: fibrinogen, GGO: ground-glass opacity. βDG: 1,3-β-D-glucan, CMV viremia: peripheral blood CMV DNA copy > 500, hypo-immunoglobin: immunoglobin G < 7 g/dL, lymphadenopathy: the shorter diameter of the lymph node ≥ 10 mm.

**Table 3:** Peripheral lymphocyte subset analysis.

Variables	ILD-PJP (*n* = 111)	Non-ILD-PJP (*n* = 205)	*t* or χ^2^ Value	*p* Value
Ly count (×10^9^/L)	818.6 ± 2295.6	693.6 ± 695.3	−0.27	0.79
Lymphocytopenia (*n*/%)	105/94.6%	192/93.7%	0.11	0.74
B cell count * (×10^9^/L)	289.0 ± 2247.0	80.1 ± 115.1	0.2	0.84
B lymphocytopenia * (*n*/%)	97/90.7%	173/91.1%	0.01	0.91
B cell percent *	13.1 ± 14.3	11.7 ± 11.3	0.61	0.54
Lower B cell percent * (*n*/%)	51(47.7)	98(51.3)	0.36	0.55
NK cell count ^#^ (×10^9^/L)	72.6 ± 128.2	64.9 ± 74.8	−0.55	0.59
NK lymphocytopenia ^#^ (*n*/%)	100/91.7%	182/92.4%	0.04	0.84
NK cell percent ^#^	13.8 ± 34.8	11.2 ± 9.9	−0.46	0.65
Lower NK cell percent ^#^ (*n*/%)	64/58.7%	108/54.6%	0.5	0.48
T cell count (×10^9^/L)	460.0 ± 449.4	546.0 ± 590.6	−0.45	0.65
T lymphocytopenia (*n*/%)	106/95.5%	185/90.2%	2.73	0.1
T cell percent	75.1 ± 15.9	75.6 ± 14.5	0.11	0.91
Lower T cell percent (*n*/%)	20/18.0%	38/18.5%	0.01	0.93
CD4^+^ T cell count (×10^9^/L)	197.5 ± 219.6	232.9 ± 281.8	−0.58	0.57
CD4^+^ T lymphocytopenia (*n*/%)	104/93.7%	184/89.8%	1.38	0.24
CD4^+^ T cell percent	31.3 ± 15.9	40.2 ± 85.3	−1.17	0.24
Lower CD4^+^ T cell percent (*n*/%)	58/52.3%	98/47.6%	0.63	0.43
CD8^+^ T cell count (×10^9^/L)	236.9 ± 266.7	277.3 ± 353.8	−0.53	0.60
CD8^+^ T lymphocytopenia (*n*/%)	92/82.9%	160/78.1%	1.04	0.31
CD8^+^ T cell percent	40.2 ± 18.4	43.5 ± 41.1	0.05	0.96
Lower CD8^+^ T cell percent (*n*/%)	13/11.7%	24/11.7%	0	0.99
CD4^+^ T/CD8^+^ T	1.3 ± 1.7	1.2 ± 1.1	−0.68	0.50

Ly: lymphocyte, PJP: Pneumocystis jirovecii pneumonia, ILD: interstitial lung disease; CD: cluster determinant. *: ILD-PJP 107 cases, non-ILD-PJP: 190, ^#^: ILD-PJP 109 cases, non-ILD-PJP: 197.

**Table 4:** The prognostic analysis from the univariable and multivariable analysis for ILD-PJP.

Variables	Univariable Analysis				Multivariable Analysis			
	χ^2^	*p*	OR	95% CI	χ^2^	*p*	OR	95% CI
ICU admission	27.7	<0.001	3.69	2.27, 6.0				
With diabetes	6.64	0.01	0.51	0.3, 0.85				
Pneumomediastinum	5.05	0.02	1.79	1.08, 2.96				
With aspergillosis	8.36	0.001	2.40	1.42, 4.06				
With HAP	13.2	<0.001	2.32	1.47, 3.66				
Respiratory failure	8.36	0.004	4.44	1.62, 12.2				
Invasive ventilation	36.1	<0.001	4.40	2.72,7.15				
Non-invasive ventilation	23.3	<0.001	3.07	1.95, 4.85	7.33	0.007	928.6	6.59, 130,796.5
Lymphocyte count	5.19	0.02	0.62	0.41, 0.93				
Lymphocyte %	9.74	0.002	0.94	0.90, 0.98				
Minimal albumin	17.3	<0.001	0.94	0.91, 0.97				
Ferritin elevation	4.20	0.04	4.45	1.07,18.51				
D-dimer	10.7	0.001	1.01	1.01,1.02	10.9	<0.001	1.2	1.08, 1.34
D-dimer elevation	8.21	0.004	3.41	1.47, 7.87				
hsCRP	6.49	0.01	1.004	1.001, 1.007				
Lymphocytopenia	4.98	0.026	1.94	1.08, 3.46				
CD4^+^ T lymphocytopenia	4.82	0.03	1.78	1.06, 2.97				
CD8^+^ T lymphocytopenia	5.12	0.02	2.22	1.11, 4.42				
Caspofungin	15.4	<0.001	2.51	1.59, 3.98				
Clindamycin	5.38	0.02	1.83	1.1, 3.07				
Primaquine	4.21	0.04	2.62	1.05, 6.56				

OR: odds ratio, PJP: Pneumocystis jirovecii pneumonia, ILD-PJP: PJP patients with pre-existing interstitial lung disease, ICU: intensive care unit, HAP: hospital-acquired pneumonia, CRP: C-reactive protein, CD: cluster determinant. Aspergillosis was defined as: ≥1 host factor criterion, 1 microbiological criterion and 1 major clinical criterion (or 2 minor criteria) according to the EORTC/MSG Consensus [22].

**Table 5:** The differences between FILD-PJP group and non-FILD-PJP group.

Variables	FILD-PJP (*n* = 70)	Non-FILD-PJP (*n* = 63)	*t* or χ^2^ Value	*p* Value
Age (y)	63.2 ± 9.5	54 ± 16.2	−3.1	0.002
Diabetes mellitus (*n*/%)	30/42.9%	14/22.2%	6.4	0.01
Invasive ventilation (*n*/%)	25/35.7%	35/55.6%	5.3	0.02
Non-invasive ventilation (*n*/%)	26/37.1%	13/20.6%	4.4	0.04
Hemoptysis (*n*/%)	9/12.9%	1/1.6%	4.5	0.03
LDH(U/L)	589.1 ± 654.2	643.5 ± 337.8	2.5	0.01
1,3-β-D-glucan (βDG, pg/mL)	592.1 ± 929.2	804.7 ± 995.9	2.6	0.01
Elevated βDG (*n*/%)	43/65.2%	53/89.8%	10.7	0.001

PJP: Pneumocystis jirovecii pneumonia, FILD-PJP: PJP patients with pre-existing fibrotic interstitial lung disease, LDH: lactate dehydrogenase.

## Data Availability

All data generated or analyzed during this study are included in this published article. Any additional data/files may be obtained from the corresponding author on reasonable request.
